# Valorization of Selected Biomass-Derived Molecules on *Olea europaea* Leaves-Biotemplated TiO_2_-g-C_3_N_4_ Photocatalysts

**DOI:** 10.3390/biomimetics9120726

**Published:** 2024-11-24

**Authors:** M. Carmen Herrera-Beurnio, Francisco J. López-Tenllado, Alejandro Ariza-Pérez, Jesús Hidalgo-Carrillo, Rafael Estevez, Juan Martín-Gómez, Francisco J. Urbano, Alberto Marinas

**Affiliations:** Departamento de Química Orgánica, Instituto Químico para la Energía y el Medioambiente (IQUEMA), Universidad de Córdoba, E-14071 Córdoba, Spain; b52hebem@uco.es (M.C.H.-B.); q82arpea@uco.es (A.A.-P.); jesus.hidalgo@uco.es (J.H.-C.); q72estor@uco.es (R.E.); q92magoj@uco.es (J.M.-G.); qo1urnaf@uco.es (F.J.U.)

**Keywords:** biotemplated photocatalysts, biomass valorization, hydrogen production, glycerol photoreforming, photoacetalization, cinnamaldehyde

## Abstract

Biotemplating technique allows the synthesis of catalysts, recreating the sophisticated structure of nature templates. In this work, some biotemplated TiO_2_ semiconductors were synthesized using *Olea europaea* leaves as templates. Then, g-C_3_N_4_ was coupled to materials to later incorporate Pt on the surface or as dopant in the structure to evaluate the efficiency of the solids in two photocatalytic applications to valorize biomass: hydrogen production through glycerol photoreforming, and photoacetalization of cinnamaldehyde with 1,2-propanediol. In glycerol photoreforming, the presence of Pt (superficial or dopant) enhanced hydrogen production, being Pt@AOLCN (a heterojunction containing biotemplated TiO_2_, g-C_3_N_4_, and Pt) the system that exhibited the highest efficiency (3053.4 µmol·g_cat_^−1^·h^−1^). For photoacetalization, while Pt reduced cinnamaldehyde conversion, it improved selectivity when incorporated on TiO_2_. Notably, carbon nitride (CN) exhibited the highest yield after 16 h of testing. The study emphasizes the importance of tailoring catalyst selection to specific reactions, as efficiency is closely tied to the structural and chemical properties of the materials. These findings contribute to the development of efficient photocatalysts for sustainable biomass valorization processes.

## 1. Introduction

In the past decades, the ‘biotemplating’ technique has allowed the synthesis of materials inspired by biological structures to recreate the sophistication, resistance, or adaptability of these compounds. In this technique, the inorganic material of interest is incorporated into the natural template, which is then removed by thermal treatments to allow the transfer of the biological structures to the inorganic ones. These biotemplated materials have been synthesized for a wide variety of applications, highlighting their use in catalysis [1]. The choice of the natural template is essential to assure obtaining materials with the desired properties [2,3]. In particular, the synthesis of photocatalysts using plants, algae, or bacteria as natural templates is of great interest as they present photosystems with high efficiency in the capture of solar radiation at both molecular and structural levels to carry out the anabolic process of photosynthesis [4]. This would allow obtaining photocatalysts with the capacity of capturing a wider range of available solar radiation.

In photocatalysis, the most used semiconductor is TiO_2_ because of its numerous advantages, such as stability, low cost, and non-toxicity, among others. There are different examples in the literature on the use of the biotemplating technique to synthesize titania photocatalysts [5,6,7,8,9]. Focusing on the use of plants and leaves as biotemplates, those of *Juncus effuses* led to a TiO_2_ used to degrade water pollutants via photocatalysis [10], whereas *Camellia* tree leaves resulted in a TiO_2_ successfully applied to CO_2_ photoreduction [11].

Another interesting field in which photocatalysis, and thus TiO_2_, is currently employed is the production of green hydrogen (H_2_) through biomass photoreforming. In this process, after the formation of the electron–hole pair by the photon capture, the hole oxidizes a compound known as a sacrificial agent that comes from biomass and acts as an electron donor, while the electrons are responsible for the reduction of protons into H_2_. This sacrificial agent allows overcoming the slow kinetics of H_2_O oxidation through water splitting and therefore permits improving the efficiency in hydrogen production reactions. Moreover, there are several studies on the effect of diverse sacrificial agents on the hydrogen production rates [12,13]. The use of glycerol as an electron donor has gained interest in recent years, as it is the main by-product of biodiesel production. Furthermore, this sacrificial agent allows high hydrogen production rates using TiO_2_ as a photocatalyst due to its easy dehydrogenation [12].

Despite its many advantages as a photocatalyst, TiO_2_ has two main drawbacks. First, it has a bandgap of around 3.2 eV, which limits its light capture to the UV range (ca. 5% of solar radiation); and second, it has a fast electron–hole pair recombination, which decreases its efficiency and active time. To address these limitations, there are different methods. One alternative is resorting to black-TiO_2_, a material that exhibits enhanced photocatalytic activity due to the narrowing of its bandgap. Nevertheless, the synthetic procedure to obtain it is rather complex as it requires hydrogenation steps at high pressure. For this catalyst, the modification of the electronic structure of TiO_2_ is caused by the introduction of Ti^3+^ defects and the presence of oxygen vacancies [14]. Other approaches include doping TiO_2_, which consists of the incorporation of metal or non-metal particles in the electronic bands of TiO_2_ to reduce the bandgap and therefore modify its absorption properties. These nanoparticles can also act as electron traps, avoiding fast recombination rates [15]. They can alternatively be deposited on the TiO_2_ surface without entering the structure. In this case, these nanoparticles capture electrons to reduce the recombination rates. Although there are different procedures to incorporate metals on the surface, photodeposition is one of the most common ones, as it uses mild conditions of pressure and temperature, and the metal can be directly incorporated in its zero-oxidation state [16]. Among metals, noble metals are the most used ones, as they exhibit a noticeable enhancement in hydrogen production, platinum being the most used. These approaches have also been used to synthesize titania through the biotemplating technique, to be applied in hydrogen production reactions. Therefore, for instance, Liu et al. [17] synthesized some titania nanotubes using cellulose as the template and photodeposited different platinum contents. These catalysts were tested for hydrogen production using methanol as a sacrificial agent, exhibiting hydrogen production rates of around 16 mmol·h^−1^·g^−1^ with the most efficient system. Cellulose nanocrystals were the chosen template for the synthesis of TiO_2_ films doped with copper [18]. Their photocatalytic hydrogen production was assessed using triethanolamine as an electron donor, observing hydrogen rates of ca. 0.73 mmol·h^−1^·g^−1^ with outstanding stability after five reuses. Another piece of research was carried out using cellulose nanocrystals to generate biotemplated TiO_2_ coupled with different metal oxides, being a system with copper the one that afforded the highest hydrogen production [19].

An alternative approach for enhancing titania photocatalytic performance is the creation of heterojunctions with another semiconductor that absorbs in the visible or in the near-infrared range [20]. In this sense, g-C_3_N_4_ has become one of the main options due to its bandgap of around 2.7–2.8 eV, its low cost, non-toxicity, or electronic and physicochemical stability. Moreover, its synthetic method is rather simple, as it can be directly obtained by thermal treatment of nitrogen-rich precursors [21]. Nevertheless, it has some disadvantages, such as fast electron–hole pair recombination, low surface area, or electron interfacial conductivity [22]. The first time that carbon nitride was reported to be an efficient photocatalyst on its own for hydrogen production with visible light was in 2009 by Wang et al. [23], where using triethanolamine as a sacrificial agent and visible light with a >420 nm cutoff filter, a hydrogen production rate of around 1 µmol·h^−1^·g_cat_^−1^ using the bare semiconductor was obtained. The formation of a g-C_3_N_4_-TiO_2_ heterojunction can lead to a higher separation rate of the photogenerated charge carriers, thus resulting in enhanced hydrogen production as compared to pure g-C_3_N_4_ [24]. g-C_3_N_4_ and TiO_2_-g-C_3_N_4_ heterojunctions have been used in several photocatalytic reactions apart from hydrogen production, such as water decontamination or CO_2_ reduction, among others [25,26,27]. Another interesting selective reaction in which g-C_3_N_4_ has been successfully applied is the photoacetalization of aldehydes with alcohols [28]. In this reaction, a hydroxyl group carries out a nucleophilic attack on the carbon of the carbonyl group, forming a hemiacetal, which, after a second analogous attack, produces an acetal and a water molecule. These acetals are high value-added products as they can be used as green solvents, pro-drugs, or pro-fragrances, hence the interest of this reaction by the scientific community. Acetalization reactions have been deeply studied in the past using homogenous or heterogenous acidic catalysis [29,30]; however, in recent years there has been a noteworthy increase in the study of this reaction through photocatalysis using UV radiation [31] or visible light. In 2016 Abdullah Khan et al. [28] studied the efficiency of g-C_3_N_4_ in the photoacetalization reaction under visible light employing different aldehydes as substrates. This paper presented g-C_3_N_4_ as a useful semiconductor for photoacetalization reactions, as the authors discovered that this material exhibited great selectivity and conversion under mild conditions of temperature and oxygen atmosphere. Later, they doped g-C_3_N_4_ with sulfur to carry out a one-pot photo-oxidation of alcohols to aldehydes and their successive photacetalization with primary alcohols such as methanol or ethanol, observing high product selectivity and reusability of the material [32]. Although, as commented above, there are many studies resorting to metals to enhance photocatalytic hydrogen production of semiconductors, in photoacetalization, the role of metals and the characteristics that the catalysts should have to ensure the highest reaction yield are still unclear.

The present work explores the valorization of biomass into catalysts and as starting materials for the photocatalytic production of other chemicals. First, we focus on the synthesis of biotemplated TiO_2_ using *Olea europaea* leaves as templates. Olive oil is of paramount importance in Andalusia. Once collected, olives are separated from olive leaves before oil extraction in the mills. Those leaves are thus an abundant by-product of the olive oil production industry; they are currently mainly burned to generate electricity. Here, we explore its valorization as biotemplates for the synthesis of a titania catalyst. This TiO_2_ will be coupled to g-C_3_N_4_ to generate heterojunctions, and then the photocatalytic efficiency of the resulting solids will be tested in two reactions of interest using biomass-derived molecules: the production of green hydrogen by glycerol photoreforming and the photoacetalization of cinnamaldehyde in the presence of 1,2-propanediol. Cinnamaldehyde is an aldehyde with a cinnamon smell widely used in fragrances, and 1,2-propanediol is an alcohol with low toxicity that can be obtained by hydrogenolysis of glycerol [33]. The reaction of both chemicals would lead to an acetal, which could be used as a pro-fragrance. Therefore, when cinnamaldehyde reacts with 1,2-propanediol, the cyclic acetal 4-methyl-2-(2-phenylvinyl)-1,3-dioxolane is formed, producing also a water molecule. During this reaction, two geometrical isomers are formed, depending on the position of the ring in cinnamaldehyde with respect to the dioxolane ring [34]. The creation of TiO_2_-g-C_3_N_4_ composites has been deeply studied in the literature, but TiO_2_ is either commercial or mainly synthesized through classical sol–gel, hydrothermal, or solvothermal methods. To the best of our knowledge, this is the first approach that resorts to biotemplated titania and to the use of TiO_2_-g-C_3_N_4_ heterojunctions to perform photoacetalization reactions. Furthermore, platinum is incorporated, both on the surface and as a dopant in the structure of the materials, to see its influence on the above-mentioned photocatalytic processes.

## 2. Materials and Methods

### 2.1. Synthesis of Photocatalysts

The biotemplated TiO_2_ system, which was denoted AOL (standing for ‘artificial olive leaf’), was synthesized adapting an experimental procedure that had already been tested by our research group [35]. Briefly, olive leaves were cut and exposed to acid hydrolysis with HCl, followed by a treatment with TiCl_3_ to promote an ion exchange between Mg^2+^ (present in the chlorophyll ring of the leaves) and Ti^3+^. Then, leaves were treated with titanium (IV) isopropoxide and subjected to reflux at 80 °C for 6 h to guarantee the formation of TiO_2_ in the structure. Afterwards, the dried solid was calcined at 550 °C for 6 h in a muffle furnace under an air atmosphere. For the synthesis of the heterojunction between AOL and g-C_3_N_4_, melamine was dissolved in distilled water and mixed with AOL in a melamine:AOL ratio of 2:1 (*w*/*w*). Subsequently, water was evaporated overnight, and the remaining solid was calcined in a semi-closed system at 550 °C for 1 h in a muffle furnace. This heterojunction was denoted AOLCN.

On the surface of AOL and AOLCN, 0.5 wt.% Pt was incorporated following a conventional photodeposition process. Adding the appropriate volume of H_2_PtCl_6_ to a dispersion of the semiconductor on a 10% (*v*/*v*) methanol solution, the slurry was irradiated for 5 h with a 125 W Hg medium pressure lamp (λ_max_ = 365 nm, 564 mW·cm^−2^). A 20 mL·min^−1^ Ar flow was maintained throughout the whole reaction, and temperature was kept at 25 °C with a water-cooling system. Solids were filtered, washed, and dried at 80 °C overnight. Samples were named Pt/AOL and Pt/AOLCN for the biotemplated TiO_2_ photocatalyst and the heterojunction with carbon nitride, respectively.

Some other systems were synthesized incorporating platinum as a dopant into AOL during the reflux step. In this sense, as platinum and titanium have similar atomic radios (135 and 140 pm, respectively), both could enter the porphyrin ring of the chlorophyll-derived compound obtained after acid hydrolysis of the olive leaves. To carry this process out, a volume of H_2_PtCl_6_ to incorporate 1 wt.% Pt was added to the reflux system. This proportion of the metal was chosen with the idea of obtaining a 0.5 wt.% Pt in the materials after adding g-C_3_N_4_ for the heterojunction. After 6 h of reflux, leaves were filtered and dried to subsequently be calcined at 550 °C for 6 h. The remaining solid was named Pt@AOL. To obtain the heterojunction with this solid, Pt@AOL and melamine were mixed following the above-mentioned procedure for AOLCN. This time, the Pt-doped heterojunction was denoted Pt@AOLCN.

Finally, g-C_3_N_4_ (CN) was synthesized by calcining melamine in a semi-closed system at 550 °C for 1 h in a muffle furnace, and platinum (0.5 wt.%) was incorporated on its surface following the same photodeposition process that was described above to obtain the solid denoted as Pt/CN.

### 2.2. Characterization of Solids

The synthesized solids were characterized by different techniques. X-Ray Diffraction analyses (XRD) were performed at the Chemical Institute for Energy and Environment (IQUEMA) of the University of Córdoba on a Bruker D8 Discover (Bruker Española S.A., Madrid, Spain) with monochromatic CuKα1 radiation (λ = 1.54 Å) over an angular range of 5–80° with a scan speed of 2.31° min^−1^. XRD diffractograms were also used to determine the crystallite size of the anatase phase, considering the full-width half-maximum of the most intense peak of anatase at a 2ϴ° value of 25.2°. Crystallite size was determined by the Scherrer equation (Equation (1)):

(1) $$D\;\left(nm\right)=\frac{K\cdot \lambda }{\beta \cdot cos\theta }$$

where *K* is the Scherrer constant (standard value of 0.9), λ is the X-ray wavelength used, β is the full-width half-maximum of the 25.2° anatase peak, and ϴ is the diffraction Bragg angle. To further study crystallinity of solids, Raman spectra were acquired by a JASCO NRS-5500 Raman instrument (Jasco Inc., Tokyo, Japan) equipped with a green laser (532 nm, power 0.7 mW) at 3 s of exposure with a CCD detector (203 K) and L1800 grating with slit setting at 100 × 1000 µm (resolution 4.25 cm^−1^). The instrument was located at the Central Service for Research Support (SCAI) of the University of Córdoba. Thermogravimetric analyses (TGA) were carried out at the University of Seville Research, Technology, and Innovation Centre (CITIUS). A TGA Discovery analyzer (TA Instruments, New Castle, DE, USA) was employed with air atmosphere at a mass flow of 100 mL·min^−1^ and a 10 °C min^−1^ ramp up to 900 °C. X-ray photoelectron spectra (XPS) were performed at the SCAI of the University of Córdoba. After being compacted on pellets, solids were outgassed to a pressure below 6 × 10^−9^ mbar at room temperature. The spectrometer was a Phoibos 150 MCD (Specs, Berlin, Germany), and it operated with AlKα (hν = 1486.6 eV) X-ray source at 400 W using C1s as reference (284.8 eV). Ti3s has a satellite in the Pt4f region [36]. Therefore, in all samples containing titania and platinum, the obtained signal for this satellite was subtracted to avoid its contribution to the signal of Pt4f. In Pt-containing solids, spectra Pt4f5/2 and 4f7/2 were fitted with a fixed spin–orbit splitting constant of 3.35 eV.

High-resolution transmission electron microscopy (HRTEM) was carried out using a ThermoScientific Talos F200i microscope (Waltham, MA, USA) at the SCAI of the University of Córdoba operating at 200 kV. Images were used to study the morphology of materials and determine the particle size distribution using ImageJ software (version 1.50i). Scanning electron microscopy (SEM) was also obtained at the SCAI with a JEOL JSM 7800F microscope (JEOL, Tokyo, Japan).

The content of platinum was analyzed by Inductively Coupled Plasma Mass Spectrometry (ICP-MS) on a Perkin Elmer Nexion350X (Waltham, MA, USA) located at the SCAI of the University of Córdoba. The spectrometer was equipped with a sample-introducing system, argon plasma ionization, and an ion-detecting quadrupole. Optical properties of solids were determined by Diffuse reflectance UV-Vis spectroscopy that was carried out on a Cary 5000 instrument from Varian (Varian Inc., Cary, NC, USA) using polytetrafluoroethylene as a white standard (100% T) and a black strip as a black reference (0% T). Bandgap values of the semiconductors were obtained by the plot of the modified Kubelka-Munk function (αhν^1/2^ for indirect transition semiconductors, or αhν^2^ for direct transition ones) versus the energy of the absorbed light (eV). Electron Paramagnetic Resonance (EPR) was carried out on a Bruker EMX micro (Bruker Española S.A, Madrid, Spain) using an X-band (9.43 GHz, 1.5 mW) microwave with a sweeping magnetic field at −173 °C. Samples were irradiated using a Newport solar simulator equipped with a 150 W Xe lamp with an optic fiber to focalize the light in the sample.

### 2.3. Photocatalytic Activity

#### 2.3.1. Hydrogen Production Through Glycerol Photoreforming

Photocatalytic efficiency of solids in hydrogen production was tested in glycerol photoreforming reactions carried out in a Penn Photoreactor m2 with UV radiation (LED light λ = 365 nm, 346 mW·cm^−2^). 1 g·L^−1^ suspensions of photocatalysts in a 10% (*v*/*v*) glycerol aqueous solution were bubbled through the whole reaction (Ar flow 4 mL·min^−1^) to maintain an inert atmosphere and were irradiated for 3 h in a 20 mL Pyrex cylindrical tube. The outlet gas phase composition was on-line analyzed by gas chromatography with thermal conductivity detector (TCD) on an Agilent Technologies 7890A gas chromatograph (Agilent Technologies, Santa Clara, CA, USA) with a Supelco Carboxen^TM^ 1010 Plot Column.

#### 2.3.2. Photoacetalization of Cinnamaldehyde with 1,2-Propanediol

Photoacetalization reactions were carried out in the same 20 mL Pyrex cylindrical tube and Penn m2 photoreactor mentioned above, but this time using visible light as an irradiation source (LED light λ = 450 nm, 1018 mW·cm^−2^) and no inert atmosphere, although the reaction was performed in a closed system. Suspensions of 1 g·L^−1^ of photocatalysts in 5 mM cinnamaldehyde (CAD) solutions in 1,2-propanediol (1,2-PDO) were irradiated for 16 h. The liquid phase composition was analyzed by gas chromatography-mass spectrometry on a single quadrupole Agilent 8890/5977C GC/MSD (Agilent Technologies, Santa Clara, CA, USA) equipped with a HP-INNOWax column. To analyze the products in the liquid phase, 0.5 mL of the reaction media were filtered, and mixed with 1 mL of a 1.33 mM dodecane (external standard) solution in propan-2-ol.

To quantify the content of acetals and cinnamaldehyde, the corresponding calibration curves were carried out. Conversion, yield and selectivity to acetals (%) of the reactions were calculated using the following equations:

(2) $$Conversion\;\left(\%\right)=\frac{\left[{CAD}_{initial}\right]-[{CAD}_{final}]}{\left[{CAD}_{initial}\right]}\times 100$$

(3) $$Yield\;\left(\%\right)=\frac{\left[Acetals\right]}{\left[{CAD}_{initial}\right]}\times 100$$

(4) $$Selectivity\;\left(\%\right)=\frac{Yield}{Conversion}\times 100\;$$

## 3. Results and Discussion

### 3.1. Characterization Results

**Figure 1:**
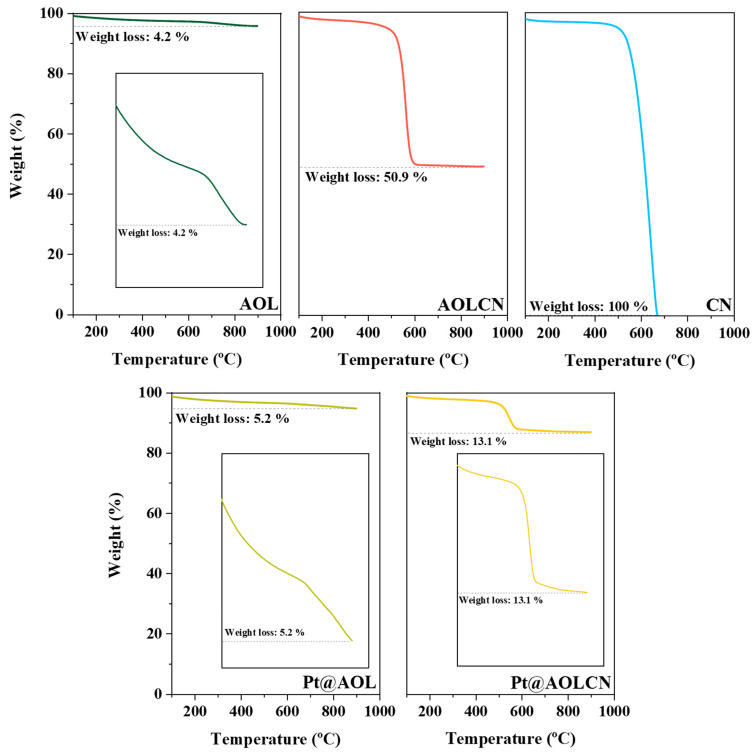
TGA results of AOL, AOLCN, CN, Pt@AOL, and Pt@AOLCN. For AOL, Pt@AOL, and Pt@AOLCN an insert was included to ease the view of weight loss.

TGA results are shown in Figure 1. AOL and Pt@AOL exhibit no significant weight loss after air calcination (ca. 4 or 5%, respectively). The weight loss observed up to 200 °C in all samples might be attributed to the evaporation of water, and at higher temperatures, to the decomposition of organic structures that form olive leaves [37,38,39]. The TG analysis of carbon nitride shows that above 600 °C the material is completely decomposed due to its instability at these temperatures [40]. Taking this fact into account, as AOLCN loses around 50% of its weight, the content of carbon nitride in this sample is around 48.7% considering the weight loss of AOL as well. Following the same reasoning, in the case of Pt@AOLCN, g-C_3_N_4_ content in the sample is around 8.3%. As Pt/AOL, Pt/AOLCN, and Pt/CN were synthesized by incorporating Pt on the surface of AOL, AOLCN, and CN, respectively, TG analyses were not carried out as same results as for the bare semiconductors were expected.

Regarding platinum content, as determined by ICP-MS (Table 1), for samples obtained by photodeposition, Pt/AOL, Pt/AOLCN, and Pt/CN, the experimental Pt content (0.39–0.47 wt.%) is close to the nominal one (0.5 wt.%). However, in the case of Pt@AOL, adding platinum during the reflux step of AOL synthesis led to an experimental incorporation of Pt of 0.45 wt.% whereas the nominal value was 1 wt.%. This suggests that photodeposition is a better option to incorporate metals, as it seems that in the synthesis of Pt@AOL, ca. 50% of the platinum was left in the liquid medium that was filtered after the reflux. A similar Pt content of ca. 0.5 wt. % was found for Pt@AOLCN.

**Figure 2:**
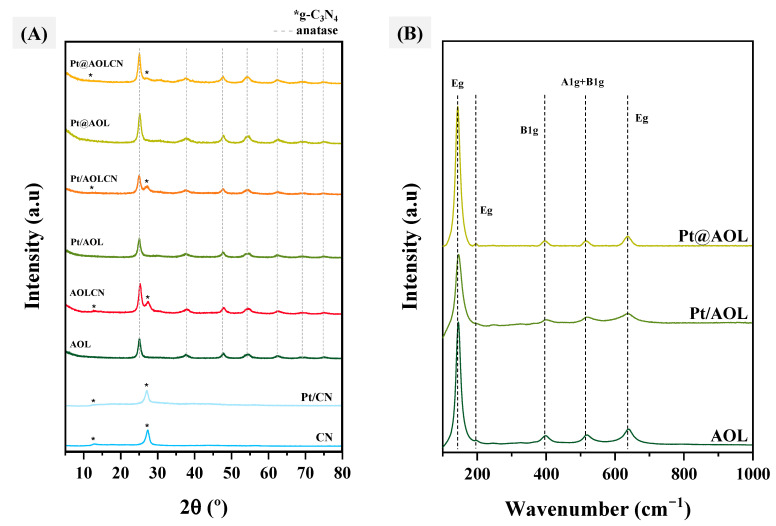
(**A**) XRD diffractograms. The asterisk refers to g-C_3_N_4_ characteristic peaks and dashed lines to anatase ones. (**B**) Raman spectra of Pt@AOL, Pt/AOL, and AOL. Carbon nitride-containing samples could not be analyzed due to fluorescence.

Figure 2A shows the diffractograms for all the materials synthesized in this work. All biotemplated samples exhibit low crystallinity, probably as a result of delayed crystallization due to impurities from the template. Looking at the diffraction pattern of AOL, the only crystallographic phase of titania is anatase (gray-dashed lines), with the corresponding reflection planes at 2ϴ° values of 25.2° (101), 37.4° (004), 47.9° (200), 54.6° (211), 62.5° (204), 69.0° (116), and 75.0° (215) [41]. This is consistent with results found in the literature where rutile appears at calcination temperatures over 600 °C [42,43]. The same anatase-corresponding peaks can be observed in all systems containing the biotemplated TiO_2_ material. In the case of AOLCN and Pt/AOLCN, two additional peaks to those of anatase are observed at 2ϴ° values of 13.0° (100) and 27.5° (002), which are attributed to the interlayer structural packing of triazine and the stacking of conjugated aromatic systems of g-C_3_N_4_, respectively [44]. In the diffractogram of Pt@AOLCN, the peak at 13.0° is not visible, while the one at 27.5° is less intense than the one present in the other heterojunctions. This is consistent with the low carbon nitride proportion that was determined by TGA (ca. 8%). The presence of anatase was further confirmed by Raman spectroscopy (Figure 2B). In these spectra, different active vibration modes for this crystallographic phase were identified at 144 cm^−1^ (Eg), 196 cm^−1^ (Eg), 398 cm^−1^ (B1g), 516 cm^−1^ (A1g + B1g), and 637 cm^−1^ (Eg), which are attributed to the symmetric stretching of Ti-O bonds in titania (Eg) and to the symmetric and antisymmetric bending (A1g and B1g) of O-Ti-O of anatase [45,46,47,48]. Carbon nitride-containing samples could not be analyzed by this technique due to the fluorescence of the materials. Crystallite size results obtained from XRD (Table 1) show anatase crystallite sizes of around 7.6–9.3 nm, which are close to those found in the literature for anatase that has been calcined at 550 °C [49,50]. As can be seen, the formation of CN on TiO_2_ structure normally leads to a slight decrease in anatase crystallite size (compare column 2 in Table 1 for AOLCN vs. AOL or Pt/AOLCN vs. Pt/AOL). However, anatase crystal size in Pt@AOLCN is slightly greater than that in Pt@AOL. This finding, together with the above-mentioned lower content in CN than expected for Pt@AOLCN (ca. 8% vs. 50%), suggests that during subsequent formation of CN from melamine on Pt@AOL to yield Pt@AOLCN, Pt catalyzes melamine decomposition exothermal process, which could be accompanied by the formation of some hotspots resulting in a slight increase in anatase crystallite size.

Bandgap values obtained from UV-Vis spectra were determined using the transformed Kubelka–Munk function plotted against the energy (eV) of the absorbed photons (Figure S1 and Table 1, fourth column). This method can be applied when semiconductors do not absorb photons from energies below their bandgap, which is the case for all the biotemplated titania solids and for carbon nitride. However, for AOLCN and Pt/AOLCN, the pigmentation of the solid (see top of Figure 6B) reflects in the absorption spectrum, which makes it difficult to determine the actual bandgap [51]. Therefore, the obtained value might not be completely accurate. However, when plotting the absorbance against the wavelength (Figure S2), it can be seen that the absorption spectra of all biotemplated samples (with and without carbon nitride) are shifted towards the visible light range. For AOL, Pt/AOL, and Pt@AOL, the onset of absorption is around 410–420 nm, which suggests that the bandgap value of all samples is similar and slightly narrower than that reported in the literature for pure anatase [52,53]. In Pt@AOLCN, two onsets are observed, one which coincides with that previously mentioned for titania systems, and another one located around 445 nm due to the interaction between titania and carbon nitride generating a heterojunction in the sample. For AOLCN and Pt/AOLCN, the onset of absorption is located at the same wavelength, and only one is visible in each sample (around 445 nm), determining the presence of the heterojunction. For CN and Pt/CN, a similar onset at around 440–450 nm is observed; however, the absorbance of Pt/CN is higher due to the pigmentation of the solid, as the presence of platinum in Pt/CN confers a grayish color to the material (see top of Figure 6B), which promotes the absorption in the visible range instead of the yellow tone of CN. This is also noticeable for the rest of the materials with platinum.

**Figure 3:**
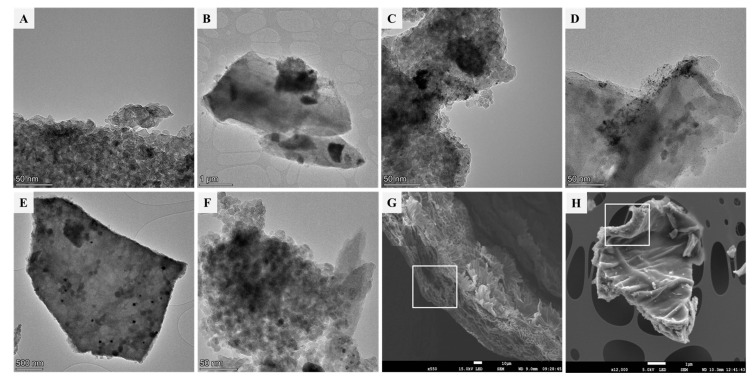
HRTEM and SEM micrographs of biotemplated systems synthesized in this work. (**A**) AOL, (**B**) AOLCN, (**C**) Pt/AOL, (**D**) Pt/AOLCN, (**E**) Pt@AOL, (**F**) Pt@AOLCN, (**G**) Fresh olive leaf, (**H**) Pt/AOL. In (**H**) the white square marks the replication of the cross section of the fresh leaf in (**G**).

Morphology and structure of materials and distribution of platinum on them were studied by HRTEM and SEM (Figure 3 and Figure S3). The structure of AOL (Figure 3A) is constituted by particles of around 10 nm, which are covered by large g-C_3_N_4_ sheets (ca. 4 µm) in AOLCN samples (Figure 3B). The same morphology can be identified for their analogous samples with platinum, both photodeposited or incorporated as dopant. Regarding this metal, photodeposition allowed the homogeneous distribution of Pt, as it is observed that in Pt/AOL, Pt/AOLCN, and Pt/CN, small 2–3 nm particles are evenly distributed throughout the surface of the semiconductors (Figure S3). Also, in Pt/AOLCN (Figure 3D), it seems that most of the metal is incorporated on the titania part of the material, which matches what our research group had already observed for TiO_2_-g-C_3_N_4_ heterojunctions synthesized with commercial titania where Pt was incorporated with UV radiation during photodeposition [16]. This might also be the reason why, according to ICP-MS, platinum content in Pt/CN is the lowest one (0.39 wt.%) within the semiconductors obtained using photodeposition to incorporate the metal, as using UV radiation does not incorporate it as efficiently. For Pt@AOL (Figure 3E and Figure S3D), where platinum was incorporated on the reflux step of the synthesis, it can be observed that the metal is aggregated, forming clusters with an average size of ca. 43 nm. This heterogeneous distribution of metal might be attributed to the temperature at which platinum was incorporated in these systems, as the reflux step was carried out at 85 °C and photodeposition was performed at 25 °C. It is possible that as high temperature increases the Brownian motion of Pt particles, the number of collisions between them rises, and therefore aggregates are formed [54]. Nevertheless, Pt@AOLCN (Figure 3F and Figure S3E) exhibits Pt particles of around 4 nm size. This might imply that the subsequent incorporation of g-C_3_N_4_ during the synthesis of this heterojunction due to the transformation of melamine resulted in Pt catalyzing the decomposition of this precursor, leading to a lower final content of carbon nitride in the sample as evidenced by TG analyses, a slight increase in anatase particles (evidenced by XRD), and result in a redispersion of platinum. Also, with scanning microscopy, the correct replication of the internal structure of the olive leaf can be seen by observing the cross section of the catalyst (Figure 3H) and the cross section of a fresh leaf (Figure 3G).

**Figure 4:**
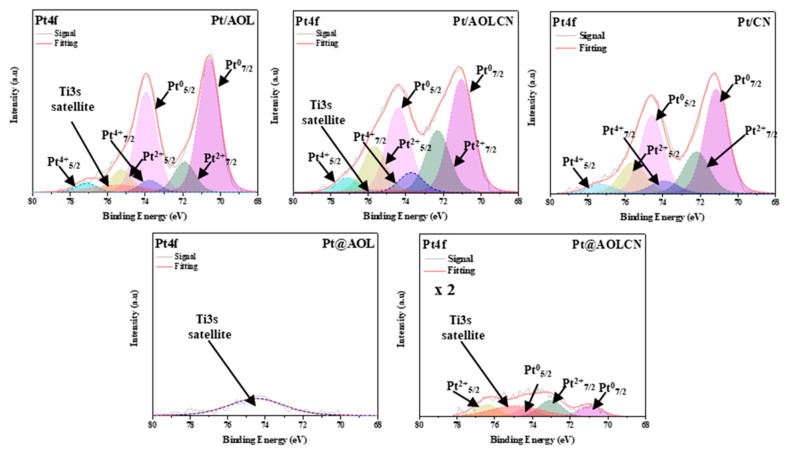
XPS of Pt4f region in platinum-containing samples.

Surface chemical composition of photocatalysts was further analyzed by XPS, and results are shown in Table 1, Figure 4 and Figures S4–11. C1s, O1s spectra were obtained for all samples, while Ti2p, N1s, and Pt4f ones were analyzed for titania, carbon nitride and platinum-containing samples, respectively. Also, for these noble metal-containing materials, Ti3s satellite spectrum in the Pt4f spectral region was measured. C1s spectra of AOL, Pt/AOL, and Pt@AOL were deconvoluted into three main peaks located approximately at 284.6 eV, 286.2 eV, and 288.3 eV, attributed to C-C of adventitious carbon, C-O, and C-C=O species, respectively [55]. O1s spectra show a peak at 529.9 eV attributed to Ti-O bonding; a second one at 531.3 eV due to the presence of OH groups; and a third one at 532.5 eV assigned to chemisorbed O_2_ by the existence of oxygen vacancies [35]. In the Ti2p spectra, there are two peaks at 458.4 eV and 464.1 eV, which are ascribed to the presence of Ti^4+^ 2p_3/2_ and Ti^4+^ 2p_1/2_, respectively. These findings show the achievement of TiO_2_ formation using the biotemplating technique [56]. Furthermore, there is a distance of 5.7 eV between each signal of the Ti2p doublet [57]. For carbon nitride-containing titania samples (AOLCN, Pt/AOLCN, and Pt@AOLCN), the same already described signals were observed in the O1s and Ti2p spectra. In Pt/CN, the signal at 529.9 eV is not present, suggesting the absence of oxygen vacancies. For C1s, three signals are identified. Apart from the one at 284.6 eV corresponding to adventitious carbon, there are two peaks at around 286 eV and 288 eV that are assigned to C-NH_2_ and N-C=N bonding in the aromatic ring, respectively [58]. In N1s spectra, four signals are observed: at 398.3 eV assigned to N-C=N bonds in triazine; at 399.5 eV due to the presence of N-(C_3_) bonds; at 400.9 eV ascribed to C-NH as a result of the incorporation of nitrogen into graphitic layers and replacement of carbon atoms; and at 404.4 eV due to the N-H bond [59,60]. The lower intensity of the N1s peak at 398.3 eV in Pt@AOLCN in comparison to that intensity in the rest of the samples can be attributed to the diminished g-C_3_N_4_ content that is present in the mentioned semiconductor.

Regarding Pt analyses (Figure 4), such metal was not found in Pt@AOL by XPS. This fact is probably due to the metal being a dopant in the structure and its heterogeneous distribution. Therefore, as XPS is a technique that analyzes the surface up to 10 nm deep, Pt could not be detected. In fact, for this sample, the signal identified at around 74.6 eV in the Pt4f spectral region might correspond to the Ti3s satellite that is located in the 70–80 eV range [36]. For Pt@AOLCN there are four signals ascribed to Pt4f_7/2_ and Pt4f_5/2_ for Pt^0^ and Pt^2+^, being higher the concentration of the latter, although the surface Pt content is lower than for the other solids. In this case, even though Pt was incorporated as a dopant, it is possible that during the synthesis of g-C_3_N_4_ on this sample, Pt redispersion also promoted its migration towards the surface of the material, being able to be identified through XPS. For Pt/AOL, Pt/AOLCN and Pt/CN, six signals are identified, corresponding to Pt4f_7/2_ and Pt4f_5/2_ for Pt^0^, Pt^2+^, and Pt^4+^. In Table 1, atomic percentages of Pt^0^, Pt^2+^, and Pt^4+^ are summarized, indicating the binding energy for Pt4f_7/2_ of each one. Pt^0^ is the main found species in these samples as a result of using UV radiation during photodeposition, which is a powerful energy source. This highlights that photodeposition is an effective procedure to incorporate metals on the surface of photocatalysts, as using Pt in its metallic state allows the transmission of photogenerated electrons from the conduction band of the catalyst onto the Fermi level of the metal, allowing a better separation of charge carriers and therefore an improved photo efficiency of the material [61]. Also, as evidenced by HRTEM, this synthetic procedure allows a homogeneous distribution of Pt. Regarding Pt/AOLCN and Pt/CN, the higher quantity of Pt^2+^ can be attributed to a higher oxidation of the sample caused by a larger period in between synthesis and analysis. Pt4f_7/2_ binding energy for Pt^0^ is 70.6 eV in Pt/AOL and 71.0 eV in Pt/AOLCN as g-C_3_N_4_ presence causes this shift towards higher binding energies (71.2 eV in Pt/CN) [16].

**Figure 5:**
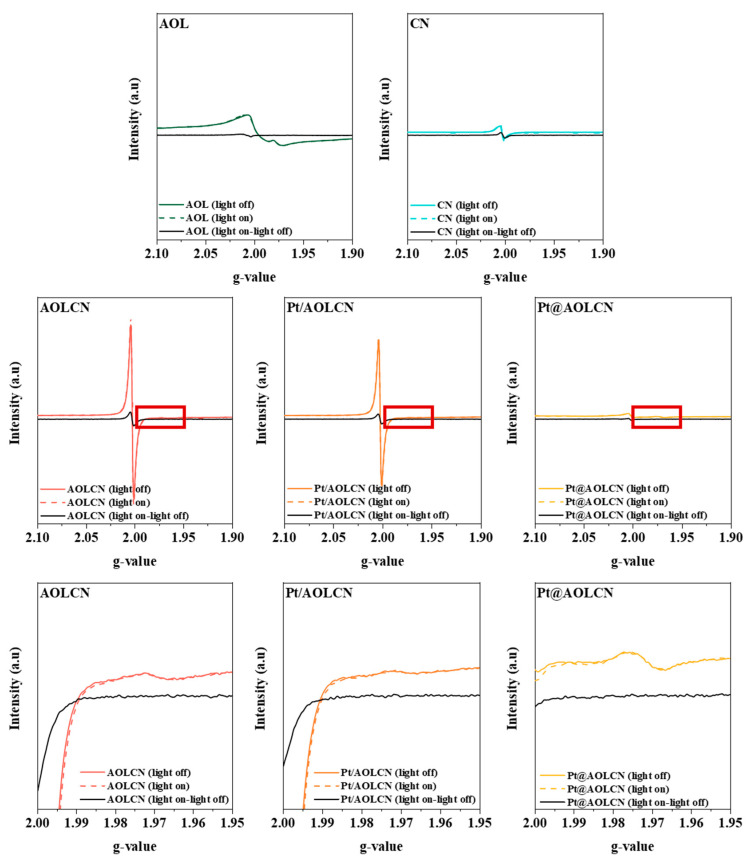
EPR spectra. The red square marks the region from g-value 2.00–1.95 that is depicted in the lower row to highlight the presence of Ti^3+^.

Electron paramagnetic resonance experiments were also carried out, and results are shown in Figure 5. All samples exhibit a peak at around g = 2.002 corresponding to a single electron [62]. The intensity of the signal of AOL is higher than that detected for P25 in the literature, probably due to a greater presence of surface defects provoked by oxygen vacancies, ascribed to g-factor values over 2 [35]. It is also possible that organic radicals resulting from some traces of organic residues from the template present in AOL could partially contribute to that signal [63,64]. In titania-containing samples, a signal at around g = 1.98 is observed, which is characteristic of Ti^3+^ species, which are electron trapping sites for photoexcited electrons in the sample [65]. The presence of such a signal both before and after irradiation of photocatalysts is evidence of the successful incorporation of Ti^3+^ in the structure of the chlorophyll-derived compound in the olive leaf. In AOLCN, Pt/AOLCN and Pt@AOLCN, focusing on the signal at g = 2.002, its intensity follows the sequence AOLCN > Pt/AOLCN >> Pt@AOLCN. Between the first two samples, the slight difference in intensity might be attributed to the presence of Pt, which acts as an electron trap after being incorporated onto the surface of the material [66]. Regarding Pt@AOLCN, one could think that the decrease in the intensity of this signal might be caused by the low carbon nitride content that is present in this heterojunction (8.3%). However, this could only justify an intensity of ca. 5 times less than in AOLCN or Pt/AOLCN. Nevertheless, results evidence a more noticeable decrease in the signal in comparison. It is possible that this effect is again caused by the platinum available in the catalyst, which, acting as an electron trap, causes the intensity to decrease.

### 3.2. Photocatalytic Production of Hydrogen

Photocatalytic efficiency of solids in hydrogen production through glycerol photoreforming was tested using 10% (*v*/*v*) glycerol aqueous solutions under UV radiation. Visible light was also tested, but hydrogen production was negligible. This is probably due to the inability of systems with AOL to absorb visible light as they are constituted by titania and to the poor adsorption of glycerol in carbon nitride-containing systems [67], which is the semiconductor that has the capacity of being excited in this range of the spectrum.

**Figure 6:**
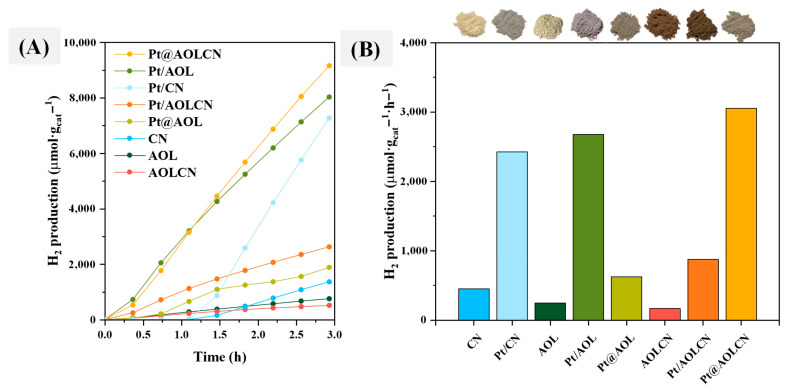
Results in hydrogen production after 3 h of irradiation under UV radiation (λ = 365 nm) in glycerol photoreforming reactions using a 10% (*v*/*v*) solution of this alcohol (**A**) Accumulated H_2_ production values for semiconductors. (**B**) Average production of hydrogen per hour for the different semiconductors. A picture of the solid is shown above each bar to show the color of the material.

Hydrogen production results are shown in Figure 6. Figure 6A shows the evolution of H_2_ production during 3 h, whereas Figure 6B represents the average hydrogen production rate on the different solids throughout the 3 h. As previously mentioned, TiO_2_ normally has a greater adsorption of glycerol and photo-efficiency under UV radiation than CN. In principle, this would explain that AOL, which is mainly constituted by titania, showed a better hydrogen production rate than AOLCN (249.7 vs. 169.3 µmol·g_cat_^−1^·h^−1^, respectively), which is formed by 50% TiO_2_ and 50% g-C_3_N_4_, approximately. Nevertheless, CN exhibited higher hydrogen production rates than both AOL and AOLCN (453.1 µmol·g_cat_^−1^·h^−1^, Figure 6B). A look at Figure 6A could give us a hint on the reason for that. Therefore, although it is evident that, as expected, CN is initially less active than AOL or AOLCN, after some induction period its activity improves significantly. The same trend is evident for Pt/CN as compared to Pt/AOL. It is possible that there is some change in the surface of g-C_3_N_4_-based samples (CN and Pt/CN) during the initial stages of reaction, which renders them more active. The fact that AOLCN produces less hydrogen than the individual semiconductors of AOL and CN might be attributed to the pigmentation of the solid, as it has a dark-brown color that may reduce the efficiency of photon arrival by making the reaction medium opaquer (see the pictures of the catalysts at the top of Figure 6B). Regardless of the Pt incorporation procedure, metal-containing materials exhibit higher hydrogen production due to the ability of Pt to capture the photogenerated electron, slowing down the electron–hole pair recombination of the material. In systems where the metal was incorporated through photodeposition, hydrogen production rate follows the sequence Pt/AOL > Pt/CN >> Pt/AOLCN (2677, 2424, 873 µmol·g_cat_^−1^·h^−1^, respectively). This difference can again be attributed to the induction period in Pt/CN and to the color of Pt/AOLCN, as mentioned with the UV-Vis spectra results. Out of all Pt-containing solids, the lowest hydrogen production rate is obtained on Pt@AOL, reaching a 626.5 µmol·g^−1^·h^−1^ production. The large Pt particle size of ca. 43.7 nm observed through HRTEM might account for that. In contrast, the metal-containing semiconductor that allows the highest production of H_2_ is Pt@AOLCN with 3053.4 µmol·g_cat_^−1^·h^−1^. In this case, this improvement could be attributed to Pt redispersion during the subsequent incorporation of melamine since, as previously mentioned, platinum might have catalyzed the calcination progress resulting in a lower content in g-C_3_N_4_ in the sample (8.3% as evidenced by TGA), a reduction in the particle size (4.1 nm), and the formation of a heterojunction between TiO_2_ and g-C_3_N_4_ around platinum particles. This good interaction among all components in the sample might be responsible for the high activity of Pt@AOLCN.

### 3.3. Photoacetalization of Cinnamaldehyde with 1,2-Propanediol

**Figure 7:**
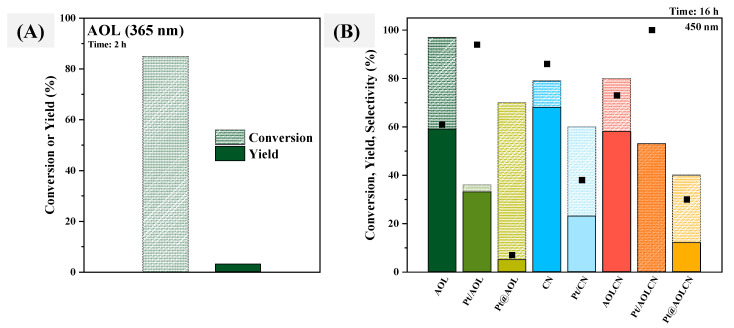
(**A**) Conversion (%, dashed column) and yield (%, full-color column) obtained for the photoacetalization of cinnamaldehyde with 1,2-propanediol after 2 h of irradiation using AOL as catalyst and UV radiation (λ = 365 nm). (**B**) Conversion (%, dashed column), yield (%, full-color column) and selectivity (black square) reached for the photoacetalization of cinnamaldehyde with 1,2-propanediol after 16 h of irradiation using visible light (λ = 450 nm).

Efficiency of solids in photoacetalization was tested using a 5 mM CAD solution utilizing 1,2-PDO as solvent. Reactions were carried out under visible light (λ = 450 nm) for several hours, and the products in the liquid phase were analyzed through GC-MS. UV radiation (λ = 365 nm) was also used as a radiation source for these reactions using AOL as catalyst. However, after 2 h of irradiation, the conversion of cinnamaldehyde in the reaction was 85% (dashed column) with a 3% yield (full-color column) to acetals (Figure 7A). These results highlight that using UV radiation is not efficient in this reaction, as CAD does not react with 1,2-PDO to selectively generate the desired acetal but instead is degraded. Therefore, the study of the efficiency of catalysts in this photoacetalization reaction was made using only visible light.

Reactions were carried out by irradiating the samples for 16 h. Blank experiments were also conducted using two different conditions: light on and no catalyst; and light off and 1 g·L^−1^ suspension of catalyst. CAD conversion after 16 h was 1% in the former and 2% in the latter, meaning that all contributions to the enhancement of conversion and yield when using a semiconductor are due to the photocatalytic process. Figure 7B shows conversion (dashed column), yield (full-color column), and selectivity (black square) for each catalyst after 16 h of irradiation. As can be seen, all platinum-containing solids exhibit lower conversions than their non-containing platinum analog. It is possible that the presence of platinum on the surface of materials blocks active titania and carbon nitride polar surface sites and therefore avoids adsorption and thus the catalytic transformation of cinnamaldehyde [68]. For biotemplated TiO_2_ systems, this fact could explain the decrease in the activity of Pt/AOL in comparison to the bare catalyst, as Pt has been incorporated on the surface through photodeposition. The higher conversion of Pt@AOL as compared to Pt/AOL could be attributed to a higher availability of -OH sites, as platinum is found in agglomerates in the bulk. For CN and Pt/CN the same tendency is observed, as well as for AOLCN, Pt/AOLCN, and Pt@AOLCN. However, it seems that selectivity is slightly linked to Pt and its position on the material, as it is evident that selectivity is higher for Pt/AOL and Pt/AOLCN (94% and 100%, respectively) having been platinum mainly incorporated on titania in both of them than for AOL (61%) and AOLCN (73%). In Pt/CN and Pt@AOLCN, where platinum is more related to carbon nitride, selectivity is lower. This may somehow suggest that when platinum is on the surface of g-C_3_N_4_, other oxidation side reactions are predominantly involved in the conversion of cinnamaldehyde.

Taking into account all the activity results that are shown in Figure 6B, it seems that CN is the most efficient system in terms of yield for photoacetalization of cinnamaldehyde with 1,2-PDO. This is consistent with that previously reported in the literature by Abdullah Khan et al. [28], where carbon nitride exhibited intrinsically high selectivity in the photacetalization of different aldehydes into acetals. Furthermore, as g-C_3_N_4_ is synthesized through a simple calcination procedure in comparison to the synthetic process of AOL or AOLCN, using g-C_3_N_4_ for this reaction would be more suitable in terms of sustainability, regarding saving in time and energy.

All in all, results obtained throughout this piece of research point out the importance of designing the appropriate catalyst for each reaction in order to obtain good photocatalytic results, as the efficiency of the systems has been proved to be different depending on their application.

## 4. Conclusions

In this work, some biotemplated TiO_2_-g-C_3_N_4_ photocatalysts were synthesized using olive leaves as templates. Also, platinum was incorporated on the materials (on the surface or as dopant) to study their photocatalytic efficiency in the photoacetalization of cinnamaldehyde with 1,2-PDO and in hydrogen production from glycerol photoreforming.

All the TiO_2_-containing semiconductors showed the characteristic peaks of the anatase phase in XRD and Raman, being also observed in those materials with carbon nitride with the typical peaks at 13° and 27.5° 2θ°. The heterojunction where Pt had been incorporated as a dopant (Pt@AOLCN) showed a lower g-C_3_N_4_ content (8.3%) by TGA, XRD, and EPR. In addition, platinum was more homogeneously distributed in it than in the Pt@AOL system (Pt size ca. 43.7 nm). XPS analysis of the Pt4f region showed 3 doublet signals in the case of the systems with photodeposited platinum, the proportion of Pt^0^ being higher due to the photodeposition method employed, and two in Pt@AOLCN; while in Pt@AOL no signal was observed for the metal, but for a satellite of Ti3s. The photocatalytic results for hydrogen production evidenced that the incorporation of Pt substantially improved this gas production, with Pt@AOLCN being the most efficient system (3053.4 µmol-g_cat_^−1^-h^−1^). The good Pt-TiO_2_-g-C_3_N_4_ interaction in the sample may account for that. However, analyzing the photoacetalization results, the systems without platinum exhibited higher conversions, probably due to a higher proportion of free active centers. Nevertheless, selectivity improved when Pt had been mainly incorporated on titania. Among the semiconductors used, CN was the one with the highest yields, demonstrating its efficiency in this reaction.

The study emphasizes the importance of synthesizing tailor-made catalysts for specific reactions, as efficiency is closely linked to their structural and chemical properties. All in all, these findings contribute to the development of efficient semiconductors to perform sustainable biomass valorization processes.

## Figures and Tables

**Table 1:** Summary of some results obtained through the characterization of photocatalysts.

Catalyst	XRD	ICP-MS	UV-Vis	XPS	
	Anatase Crystallite Size (nm)	Pt Content (%)	Bandgap (eV)	Pt4f (eV, At. %)	Surface Pt wt.%
CN	-	^a^	2.82	^a^	^a^
AOL	8.8	^a^	3.06	^a^	^a^
AOLCN	7.8	^a^	2.60	^a^	^a^
Pt/CN	-	0.39	2.81	71.2, 67.4 (Pt^0^)	0.66
				72.3, 22.8 (Pt^2+^)	
				73.8, 9.8 (Pt^4+^)	
Pt/AOL	8.5	0.47	3.06	70.6, 75.7 (Pt^0^)	0.73
				71.9, 17.3 (Pt^2+^)	
				73.8, 7.0 (Pt^4+^)	
Pt/AOLCN	7.6	0.45	2.60	71.0, 57.9 (Pt^0^)	0.63
				72.3, 31.8 (Pt^2+^)	
				73.7, 10.3 (Pt^4+^)	
Pt@AOL	8.5	0.45	3.01	^b^	^b^
Pt@AOLCN	9.3	0.53	3.02 (AOL)	70.9, 39.9 (Pt^0^)	0.40
			2.76 (Heterojunction)	74.3, 60.1 (Pt^2+^)	

^a^ Pt non-containing samples; ^b^ Not detected.

## Data Availability

Data will be available on request.

## Supplementary Materials

The following supporting information can be downloaded at: https://www.mdpi.com/article/10.3390/biomimetics9120726/s1, Figure S1: Bandgap value of semiconductors.; Figure S2: Absorbance spectra of samples determined by UV-Vis spectroscopy.; Figure S3: HRTEM micrographs of platinum-containing solids; Figure S4: XPS of CN.; Figure S5: XPS of AOL.; Figure S6: XPS of AOLCN.; Figure S7: XPS of Pt/CN.; Figure S8: XPS of Pt/AOL.; Figure S9: XPS of Pt@AOL; Figure S10: XPS of Pt/AOLCN.; Figure S11: XPS of Pt@AOLCN.
