# Supplementary material for: Valorization of Selected Biomass-Derived Molecules on Olea europaea Leaves-Biotemplated TiO2-g-C3N4 Photocatalysts
Source: Biomimetics (Basel). 2024 Nov 24;9(12):726. doi: 10.3390/biomimetics9120726 (PMC11672941; doi:10.3390/biomimetics9120726)
Supplement: Supplementary file 1 [file biomimetics-09-00726-s001.zip › biomimetics-3304747-supplementary.pdf]

Supplementary Information

Valorization of Selected Biomass-Derived Molecules on *Olea Europaea* Leaves-Biotemplated TiO_2-g-C_3N_4 Photocatalysts

M. Carmen Herrera-Beurnio, Francisco J. López-Tenllado*, Alejandro Ariza-Pérez, Jesús Hidalgo-Carrillo, Rafael Estevez, Juan Martín-Gómez, Francisco J. Urbano, Alberto Marinas*

Departamento de Química Orgánica, Instituto Químico para la Energía y el Medioambiente (IQUEMA), Universidad de Córdoba, E-14071 Córdoba, Spain

*Corresponding authors. E-mail addresses: b42lotef@uco.es (Francisco J. López-Tenllado), qo2maara@uco.es (Alberto Marinas)

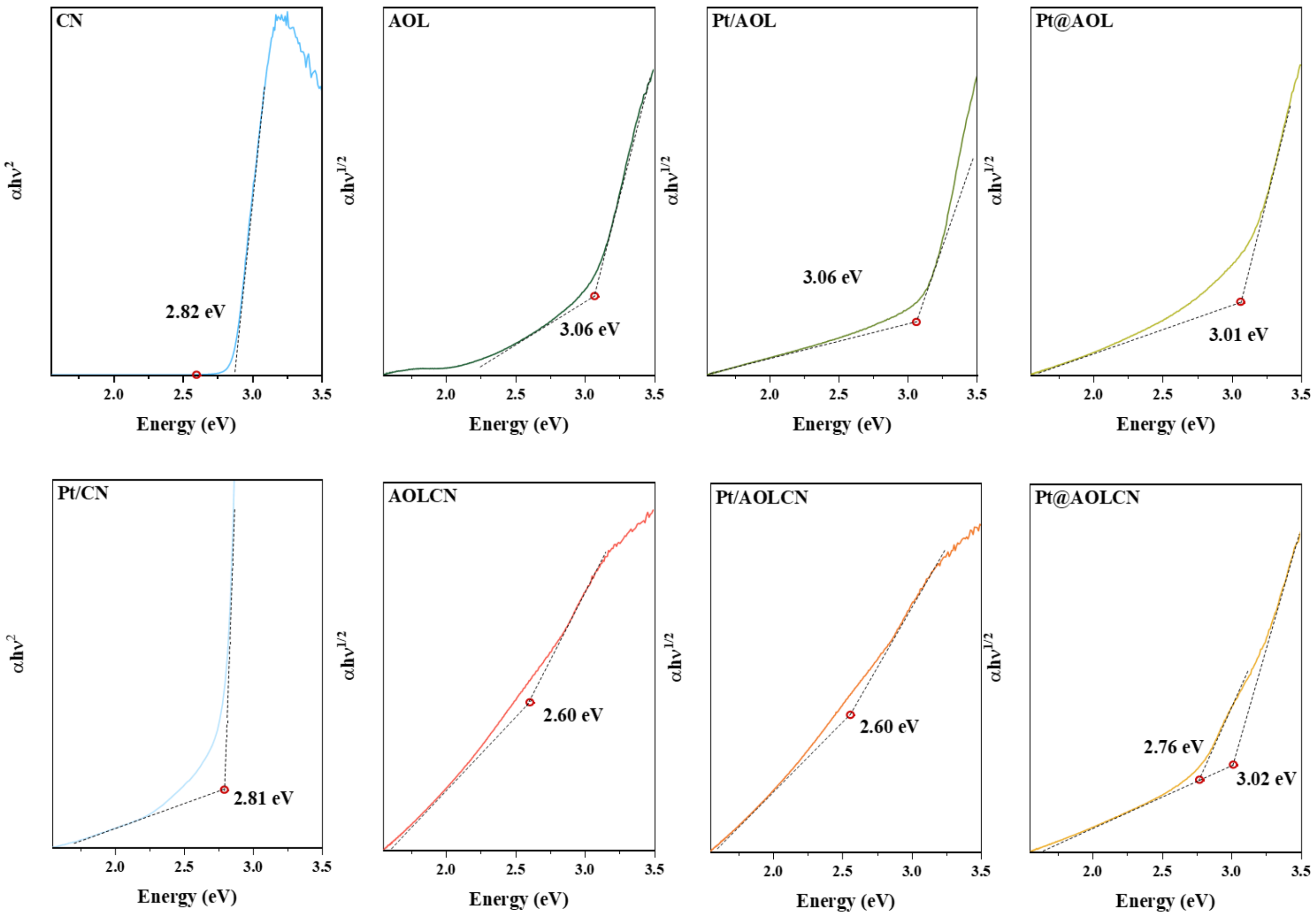

Figure S1. Bandgap value of semiconductors calculated plotting the $(\alpha h\nu)^{1/2}$ for indirect transition bandgaps or $(\alpha h\nu)^2$ for direct transition ones, calculated by the modified of the Kubelka-Munk function against the energy (eV).

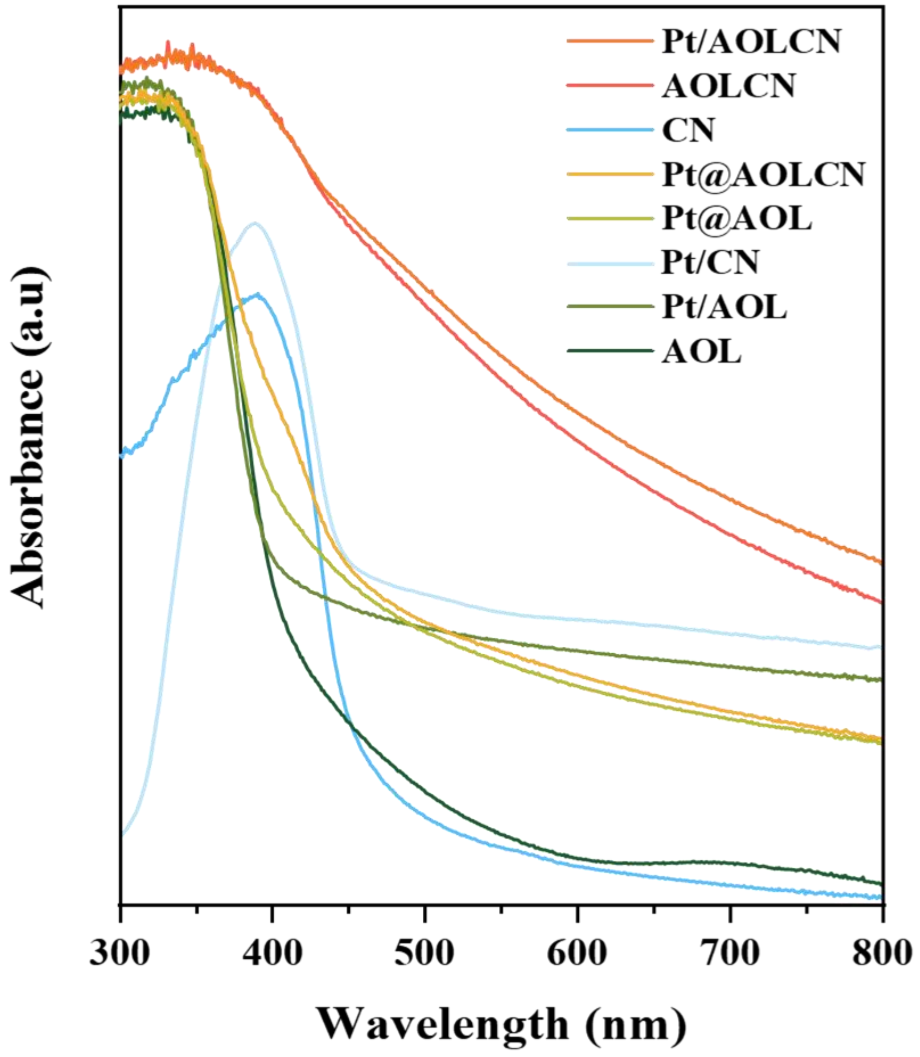

Figure S2. Absorbance spectra of samples determined by UV-Vis spectroscopy.

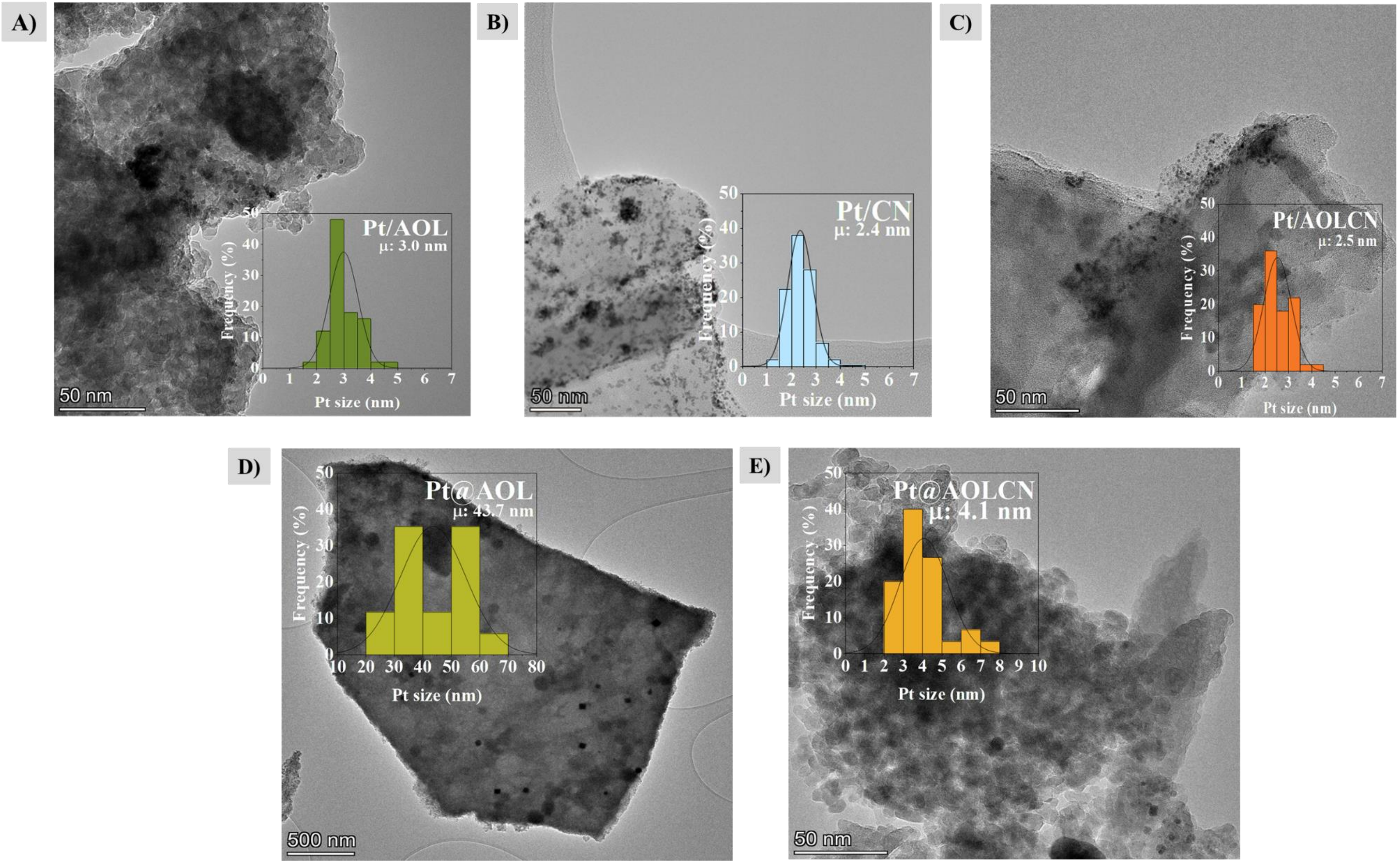

Figure S3. HRTEM micrographs of platinum-containing solids. Pt particle size histogram is also depicted, showing the average platinum particle size.

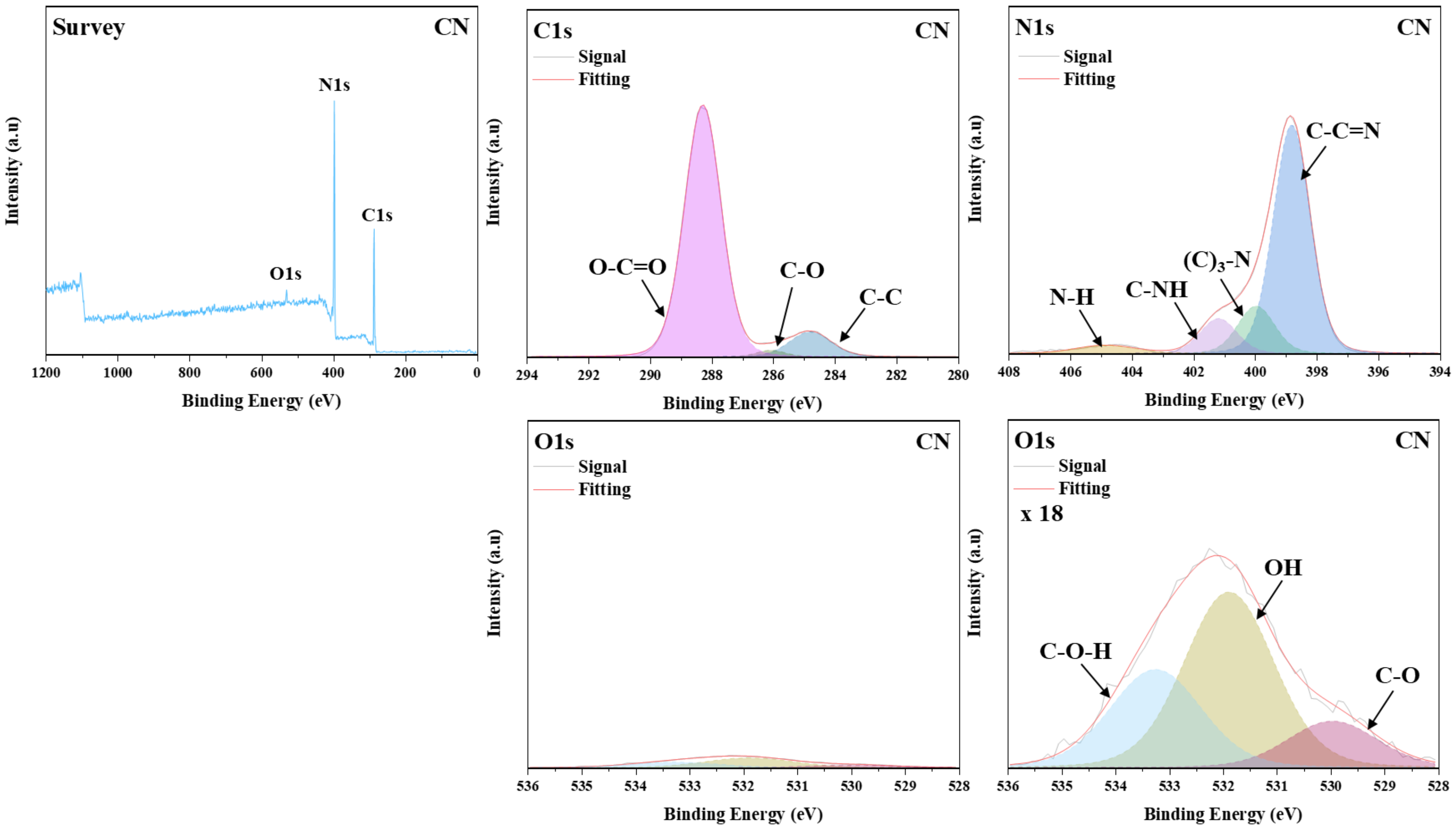

Figure S4. XPS spectra of CN. The intensity of signal was adjusted for all semiconductors, in order to compare the graphics among them.

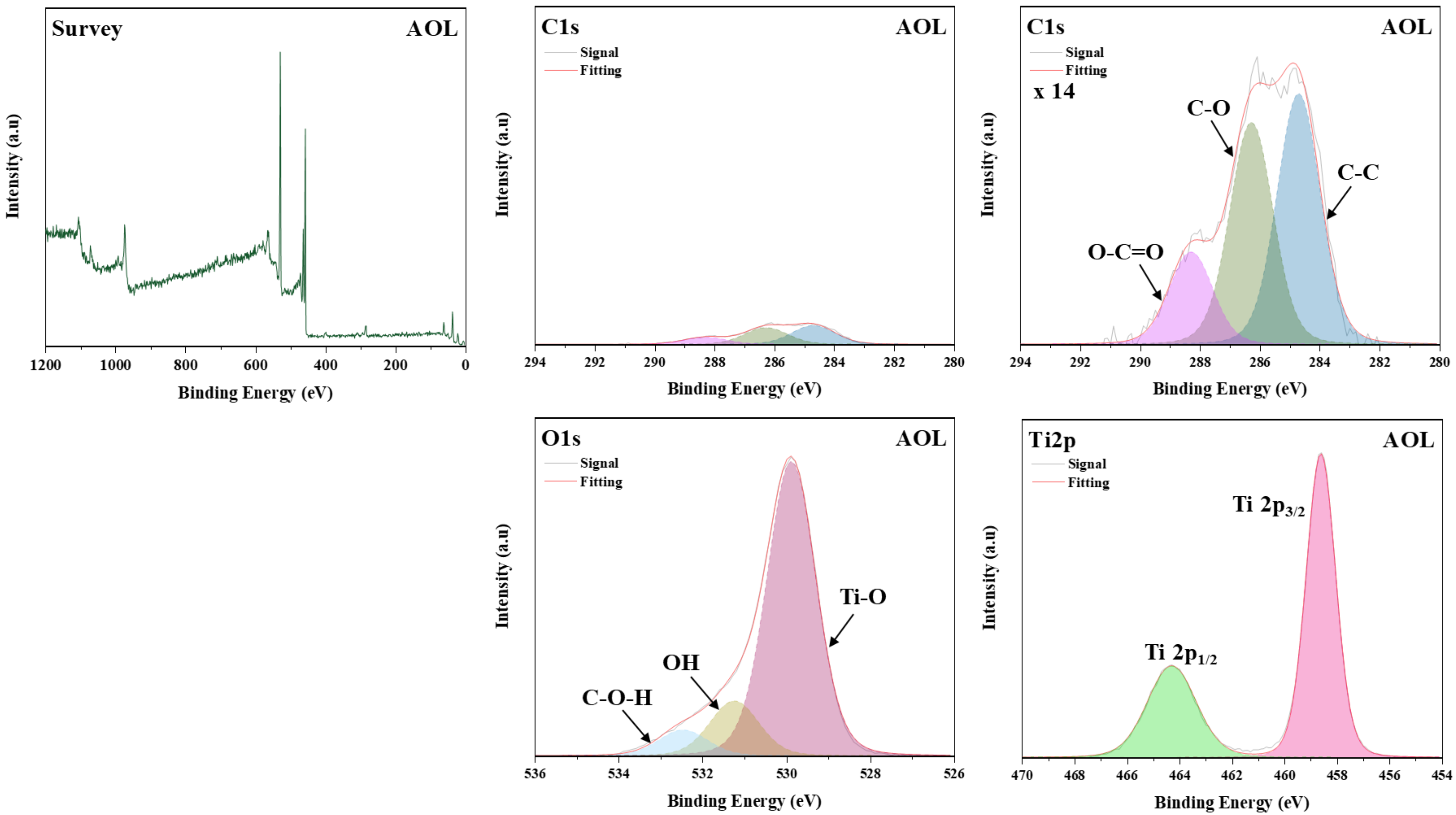

Figure S5. XPS spectra of AOL. The intensity of signal was adjusted for all semiconductors, in order to compare the graphics among them.

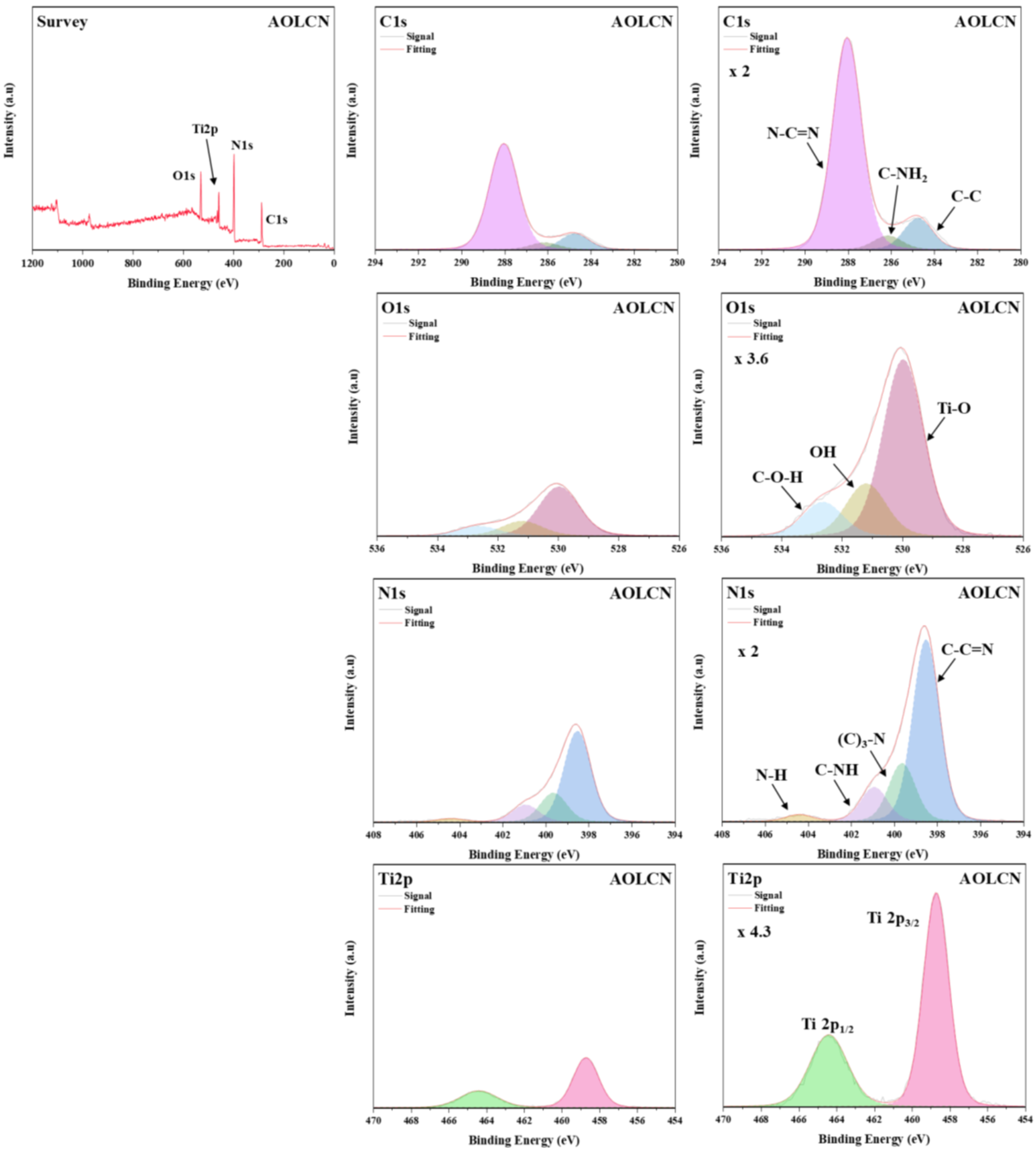

Figure S6. XPS spectra of AOLCN. The intensity of signal was adjusted for all semiconductors, in order to compare the graphics among them.

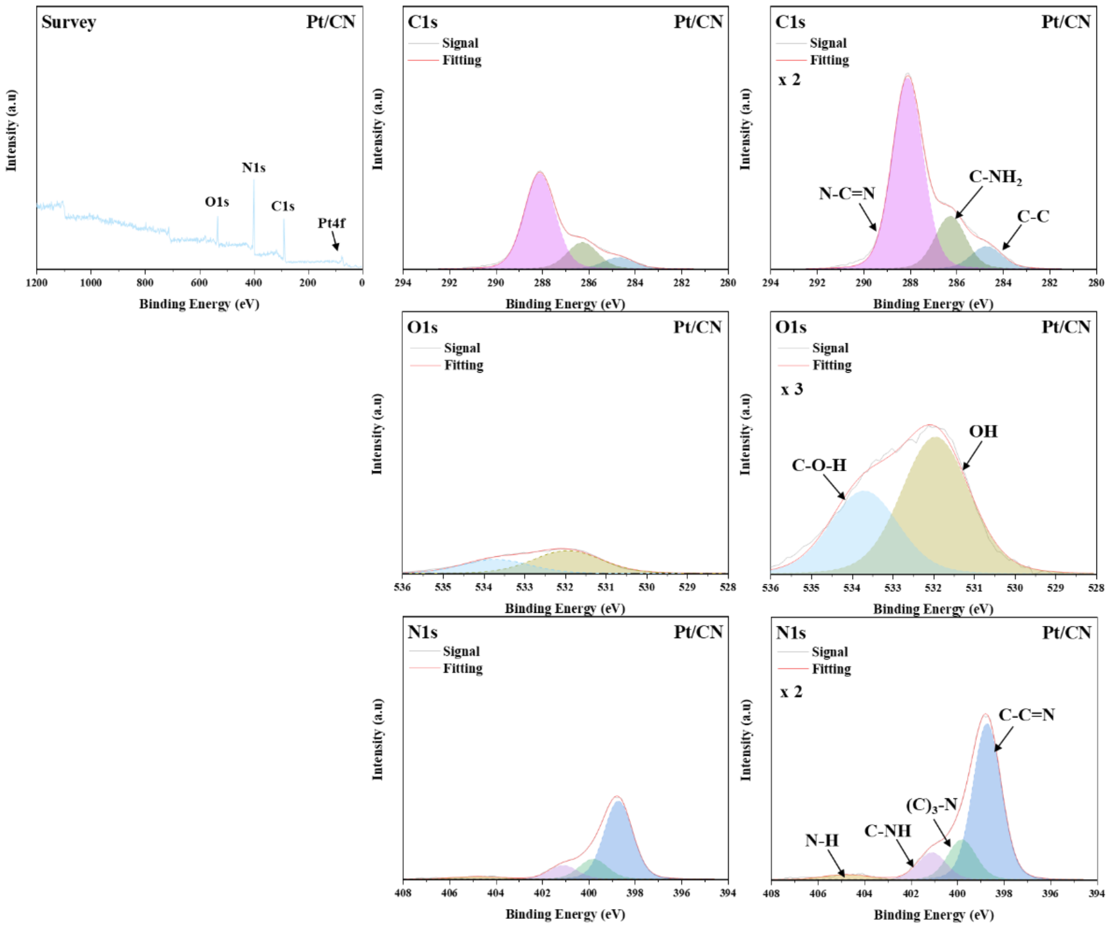

Figure S7. XPS spectra of Pt/CN. The intensity of signal was adjusted for all semiconductors, in order to compare the graphics among them. Pt4f region is shown in Figure 3 in the main text.

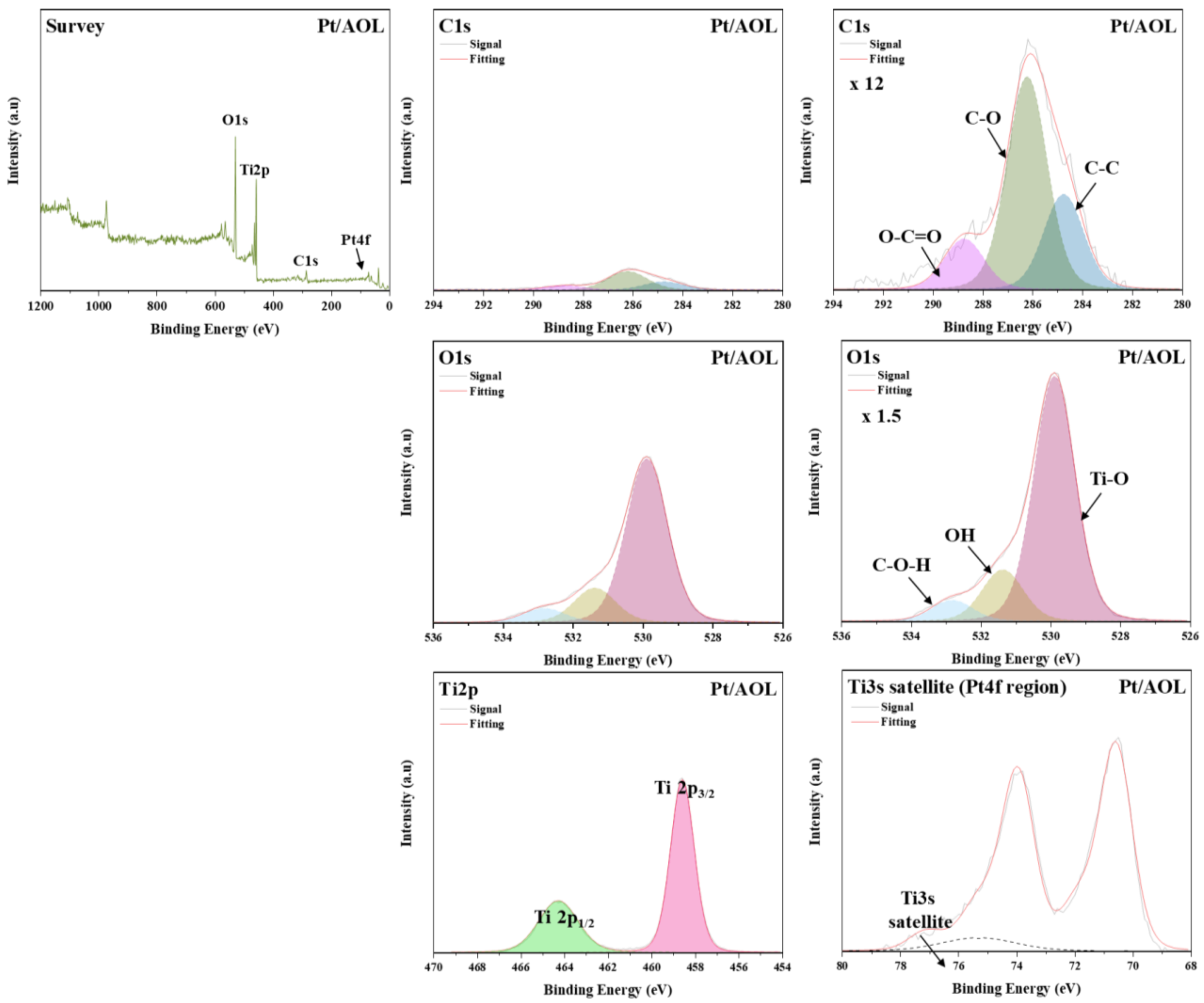

Figure S8. XPS spectra of Pt/AOL. The intensity of signal was adjusted for all semiconductors, in order to compare the graphics among them. Pt4f region is shown in Figure 3 in the main text.

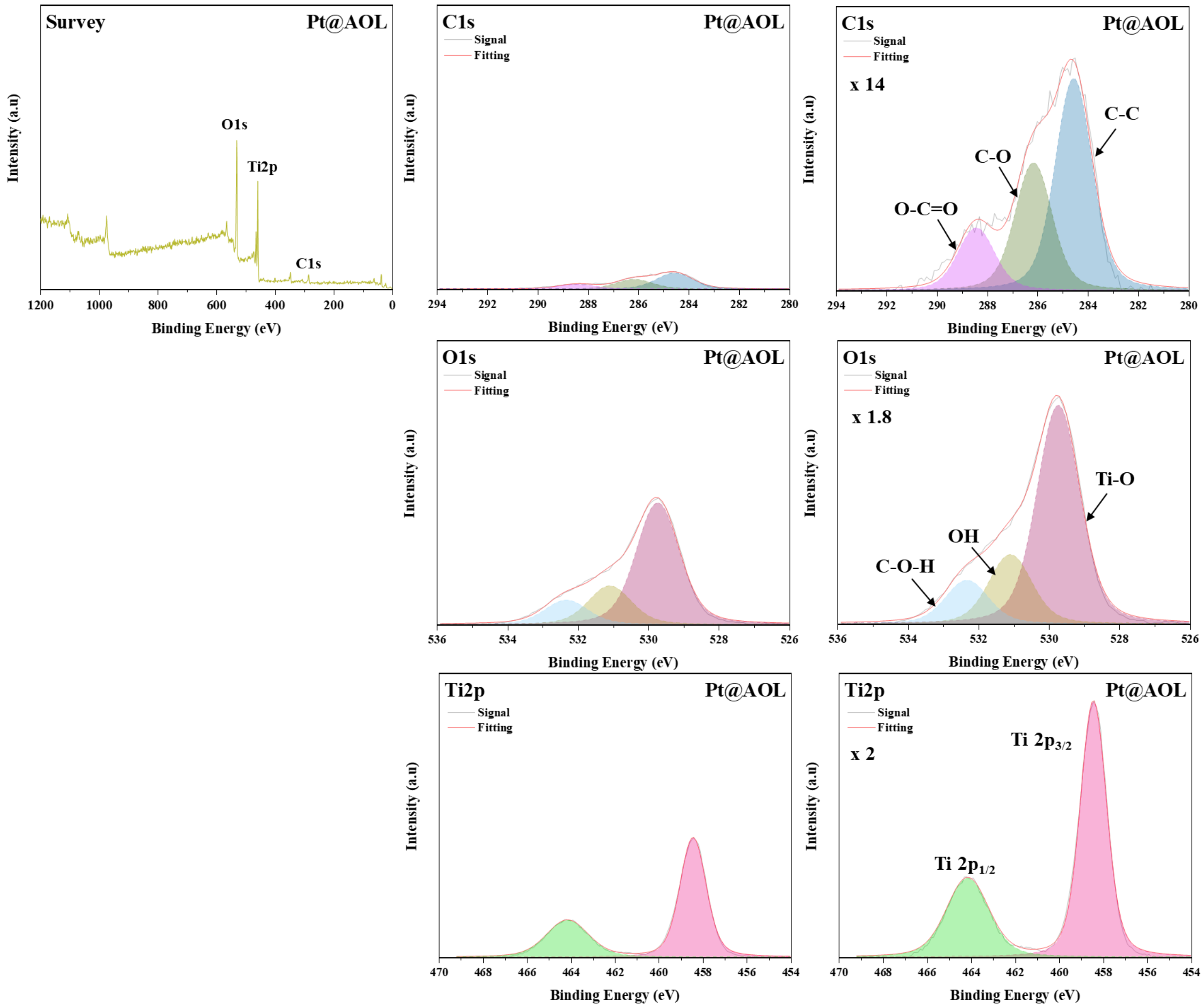

Figure S9. XPS spectra of Pt@AOL. The intensity of signal was adjusted for all semiconductors, in order to compare the graphics among them. Pt4f region is shown in Figure 3 in the main text.

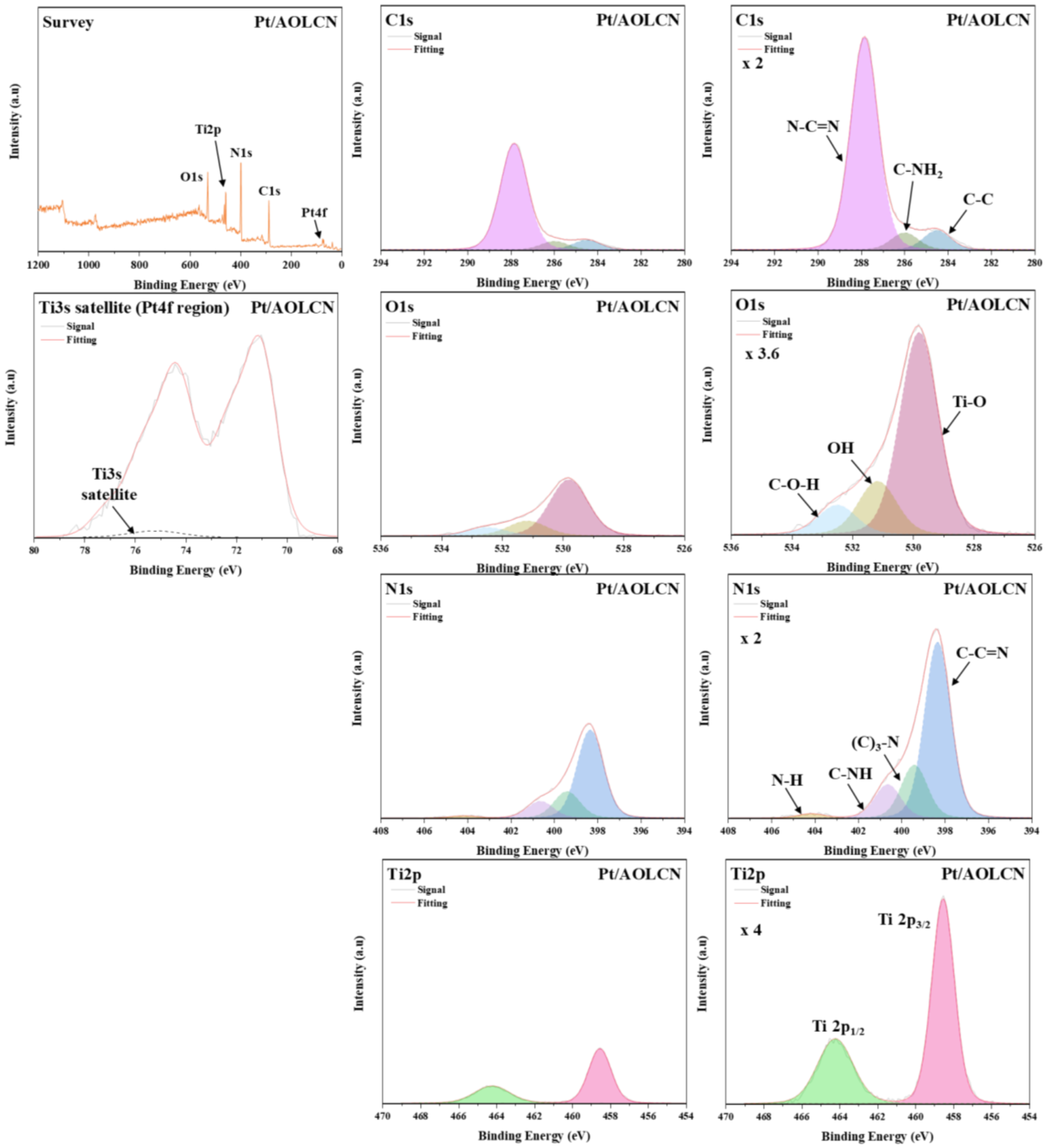

Figure S10. XPS spectra of Pt/AOLCN. The intensity of signal was adjusted for all semiconductors, in order to compare the graphics among them. Pt4f region is shown in Figure 3 in the main text.

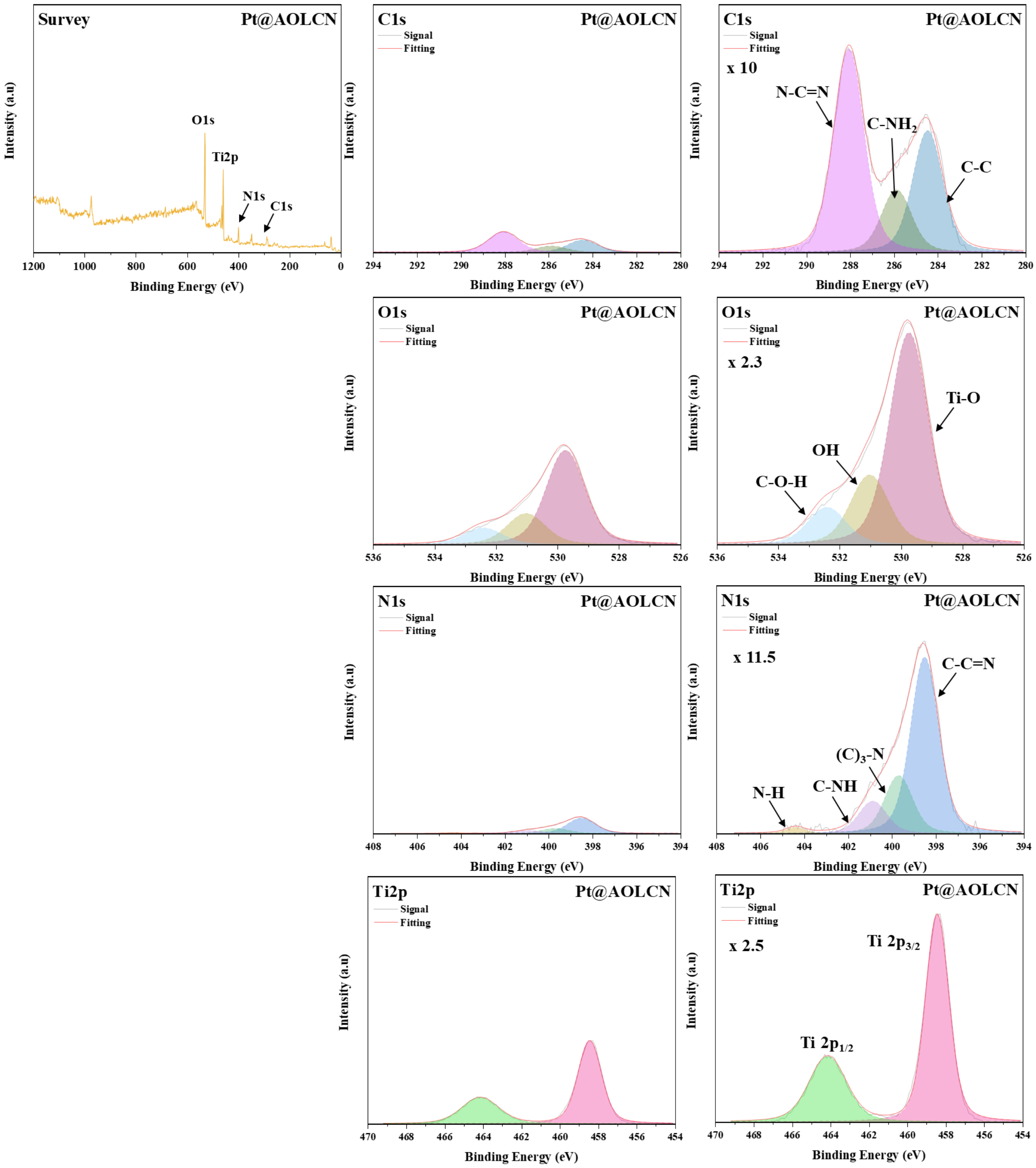

Figure S11. XPS spectra of Pt@AOLCN. The intensity of signal was adjusted for all semiconductors, in order to compare the graphics among them. Pt4f region is shown in Figure 3 in the main text.
